# Global analysis of actionable genomic alterations in thyroid cancer and precision-based pharmacogenomic strategies

**DOI:** 10.3389/fphar.2025.1524623

**Published:** 2025-04-14

**Authors:** Samantha Espinoza-Ferrao, Gabriela Echeverría-Garcés, Sebastián Rivera-Orellana, José Bueno-Miño, Emilia Castellanos-Molina, Melanie Benítez-Núñez, Andrés López-Cortés

**Affiliations:** ^1^ Cancer Research Group (CRG), Faculty of Medicine, Universidad de Las Américas, Quito, Ecuador; ^2^ Centro de Referencia Nacional de Genómica, Secuenciación y Bioinformática, Instituto Nacional de Investigación en Salud Pública “Leopoldo Izquieta Pérez”, Quito, Ecuador; ^3^ Latin American Network for the Implementation and Validation of Clinical Pharmacogenomics Guidelines (RELIVAF-CYTED), Santiago, Chile

**Keywords:** thyroid cancer, pathogenic variants, allele frequencies, drug prescription, global populations, pharmacogenomic testing, precision oncology

## Abstract

**Introduction:**

Thyroid cancer, a prevalent endocrine malignancy, has an age-standardized incidence rate of 9.1 per 100,000 people and a mortality rate of 0.44 per 100,000 as of 2024. Despite significant advances in precision oncology driven by large-scale international consortia, gaps persist in understanding the genomic landscape of thyroid cancer and its impact on therapeutic efficacy across diverse populations.

**Methods:**

To address this gap, we performed comprehensive data mining and *in silico* analyses to identify pathogenic variants in thyroid cancer driver genes, calculate allele frequencies, and assess deleteriousness scores across global populations, including African, Amish, Ashkenazi Jewish, East and South Asian, Finnish and non-Finnish European, Latino, and Middle Eastern groups. Additionally, pharmacogenomic profiling, *in silico* drug prescription, and clinical trial data were analyzed to prioritize targeted therapeutic strategies.

**Results:**

Our analysis examined 56,622 variants in 40 thyroid cancer-driver genes across 76,156 human genomes, identifying 5,001 known and predicted oncogenic variants. Enrichment analysis revealed critical pathways such as MAPK, PI3K-AKT-mTOR, and p53 signaling, underscoring their roles in thyroid cancer pathogenesis. High-throughput validation strategies confirmed actionable genomic alterations in RET, BRAF, NRAS, KRAS, and EPHA7. Ligandability assessments identified these proteins as promising therapeutic targets. Furthermore, our findings highlight the clinical potential of targeted drug inhibitors, including vandetanib, dabrafenib, and selumetinib, for improving treatment outcomes.

**Discussion:**

This study underscores the significance of integrating genomic insights with pharmacogenomic strategies to address disparities in thyroid cancer treatment. The identification of population-specific oncogenic variants and actionable therapeutic targets provides a foundation for advancing precision oncology. Future efforts should focus on including underrepresented populations, developing population-specific prevention strategies, and fostering global collaboration to ensure equitable access to pharmacogenomic testing and innovative therapies. These initiatives have the potential to transform thyroid cancer care and align with the broader goals of personalized medicine.

## Introduction

Thyroid cancer is among the most commonly diagnosed endocrine malignancies, characterized by abnormal cellular growth within the thyroid gland (Xing, 2013). Its development is influenced by various factors, including hormonal imbalances, genetic predisposition, ethnicity, environmental exposures, epigenetic modifications, driver mutations, and dysregulation of protein expression and signaling pathways (Hanahan, 2022). Despite its high incidence rate, thyroid cancer generally exhibits relatively low mortality. According to the World Health Organization (WHO) and Global Cancer Statistics (GLOBOCAN), the global age-standardized incidence rate of thyroid cancer is 9.1 per 100,000 inhabitants, with an age-standardized mortality rate of 0.44 per 100,000 (Bray et al., 2024).

Advances in genomics, particularly following the Human Genome Project in 1990 (Green et al., 2020; Nurk et al., 2022), have significantly enhanced our understanding of the genetic and molecular mechanisms underlying thyroid cancer. Modern sequencing technologies have been instrumental in identifying cancer driver genes (Kandoth et al., 2013; Lawrence et al., 2014), germline variants (Lu et al., 2015), cancer-driving mutations in both coding and non-coding regions (Sjöblom et al., 2006; Tamborero et al., 2013; Porta-Pardo et al., 2017; Rheinbay et al., 2020), druggable enzymes (Rubio-Perez et al., 2015), drug resistance genes (Vasan et al., 2019), and pharmacogenomic annotations (Quinones et al., 2014; López-Cortés et al., 2020c; 2017; Varela et al., 2021). Moreover, artificial intelligence predictions (López-Cortés et al., 2020a; Jumper et al., 2021; López-Cortés et al., 2024; Cabrera-Andrade et al., 2020a) have contributed to identifying novel therapeutic targets associated with thyroid cancer progression.

Despite these advancements, treatment responses among thyroid cancer patients remain highly variable (Raguz and Yagüe, 2008; Mansoori et al., 2017). Precision oncology provides a promising approach to address this variability by tailoring therapies to individual patients based on their specific genomic alterations and clinical data (Quinones et al., 2014; Garraway et al., 2013). This approach has already facilitated the development of targeted therapies, such as inhibitors for *BRAF* and *RET* mutations, which are commonly implicated in thyroid cancer (Espinosa et al., 2007; Salvatore et al., 2021). However, patient heterogeneity and variable outcomes highlight the need for further refinement of these strategies.

Thyroid cancer also demonstrates significant differences in incidence, progression, and outcomes across ethnic groups, suggesting a complex interplay between genetic predisposition and environmental factors (Bhattacharya et al., 2023; Özdemir and Dotto, 2017). These disparities underscore the importance of population-specific genomic studies to ensure equitable access to precision medicine (López-Cortés et al., 2020c). However, the underrepresentation of diverse populations in cancer research limits the generalizability and applicability of existing findings on a global scale (Guerrero et al., 2018; García-Cárdenas et al., 2024). This gap poses significant challenges to developing inclusive pharmacogenomic strategies and precision oncology frameworks. To address these challenges, we performed *in silico* analyses to identify actionable genomic variants—alterations with potential therapeutic relevance that are associated with pathways or mechanisms amenable to pharmacological or experimental interventions in thyroid cancer. Furthermore, we assessed the prevalence of these variants across different populations and prioritized therapeutic approaches aligned with the principles of precision medicine.

## Methods

### Incidence and mortality of thyroid cancer

The Global Cancer Observatory (https://gco.iarc.fr/) allows for a comprehensive evaluation of the global cancer burden. Using the most recent version of GLOBOCAN, we have retrieved and ranked the countries worldwide with the highest estimated age-standardized incidence and mortality rates for thyroid cancer (Bray et al., 2024).

### Thyroid cancer driver genes

The intOGen framework (https://www.intogen.org) is a tool that identifies cancer genes and determines their mechanism of action across different types of tumors (Martínez-Jiménez et al., 2020). The current version of the intOGen pipeline uses seven methods to identify cancer driver genes based on point mutations: dNdScv (Martincorena et al., 2017), CBaSE (Weghorn and Sunyaev, 2017), MutPanning (Dietlein et al., 2020), OncodriveCLUSTL (Arnedo-Pac et al., 2019), HotMAPS (Tokheim et al., 2016), smRegions (Martínez-Jiménez et al., 2020), and OncodriveFML (Mularoni et al., 2016). Therefore, we retrieved 40 thyroid cancer driver genes and identified their involvement as oncogenes (Sondka et al., 2018), tumor suppressor genes (Sondka et al., 2018), kinase genes (Manning et al., 2002; Eid et al., 2017), DNA-repair genes (Lange et al., 2011; Wood et al., 2001), RNA-binding proteins (Hentze et al., 2018), cell cycle genes (Bar-Joseph et al., 2008), metastatic genes (Zheng et al., 2018), and cancer immunotherapy genes (Patel et al., 2017).

### Identification of the oncogenic variome

The identification of the thyroid oncogenic variome was divided into two steps. In the first step, we extracted 56,622 single nucleotide and insertion/deletion variants belonging to 40 thyroid cancer driver genes from the Genome Aggregation database (gnomAD v3.2.1) (https://gnomad.broadinstitute.org/), using the complete sequence of a human genome (GRCh38/hg38) as the reference genome (Collins et al., 2020; Karczewski et al., 2020; Nurk et al., 2022). In the second step, we performed the OncodriveMUT and boostDM methods integrated into the Cancer Genome Interpreter platform (https://www.cancergenomeinterpreter.org) to assess the tumorigenic potential of the 56,622 aforementioned genomic variants (Muiños et al., 2021; Tamborero et al., 2018). OncodriveMUT is a developed rule-based approach that combines genomic features such as clusters of somatic mutations, regions depleted by germline variants, gene mechanism of action, and gene signals of positive selection, whereas boostDM is a machine learning-based methodology for *in silico* saturation mutagenesis of cancer genes to assess the oncogenic potential of mutations in human tissues. Both methods let us classify driver variants into known, predicted, and passenger mutations using the Catalog of Validated Oncogenic Mutations (Muiños et al., 2021; Tamborero et al., 2018).

### Deleteriousness score of the oncogenic variome

Combined Annotation-Dependent Depletion (CADD) version 1.4 (https://cadd.gs.washington.edu/) is an integrative annotation built from more than 60 genomic features that measure the deleteriousness of single nucleotide and insertion/deletion variants in the human genome (Kircher et al., 2014). This framework is adapted to the GRCh38/hg38 human reference genome and integrates multiple annotations into one metric by contrasting variants that survived natural selection with simulated mutations (Rentzsch et al., 2019). In this study, we calculated the CADD score for ranking the deleteriousness of the known and predicted oncogenic variome located in thyroid cancer driver genes. The deleteriousness of the oncogenic variome was categorized according to its CADD score in very high (30–50), high (25–30), medium (15–25), low (10–15), and very low (0–10).

### Protein-protein interactome network

To better understand the connectivity among thyroid cancer driver proteins, we constructed a protein-protein interactome (PPi) network. This analysis utilized human proteome data obtained through the Cytoscape StringApp, focusing on high-confidence interactions (cutoff = 0.9) based on experimental evidence (Szklarczyk et al., 2015; Shannon et al., 2003; Doncheva et al., 2019). The analysis included all thyroid cancer driver proteins identified in the human proteome using the intOGen pipeline and the Catalogue of Somatic Mutations in Cancer (COSMIC) - Cancer Gene Census (CGC) database (Martínez-Jiménez et al., 2020; Sondka et al., 2018). To characterize the network, we calculated degree centrality, which quantifies the number of edges connected to each node within the network (López-Cortés et al., 2018; López-Cortés et al., 2021b; López-Cortés et al., 2022a; Cabrera-Andrade et al., 2020b). These calculations were performed using the CytoNCA app (Tang et al., 2015). For improved organization and visualization, the nodes and edges were arranged using the degree-sorted circle layout. The PPi network was then visualized with Cytoscape software v.3.10 (Szklarczyk et al., 2015; Shannon et al., 2003). Finally, the degree centrality analysis offered valuable insights into the network properties of thyroid cancer driver proteins, shedding light on their roles within the interaction network.

### Functional enrichment analysis

The enrichment analysis gives scientists curated interpretation of gene/protein sets from omics-scale experiments (López-Cortés et al., 2020b; López-Cortés et al., 2022b; López-Cortés et al., 2021a). In this context, we performed a functional enrichment analysis of thyroid cancer driver genes/proteins that carry known and predicted oncogenic variants by using g:Profiler version e101_eg48_p14_baf17f0 (https://biit.cs.ut.ee/gprofiler/gost) (Raudvere et al., 2019) to obtain significant annotations (Benjamini–Hochberg, false discovery rate (FDR) *q* < 0.001) related to gene ontology (GO) biological processes (The Gene Ontology Consortium, 2021), Kyoto Encyclopedia of Genes and Genomes (KEGG) signaling pathways (Kanehisa and Goto, 2000), Reactome signaling pathways (Fabregat et al., 2016), Wikipathways (Slenter et al., 2018), and human phenotype ontology (HP) (Köhler et al., 2021). Lastly, significant annotations related to signaling pathways and thyroid cancer were manually curated and visualized through a Manhattan plot.

### Allele frequencies across human populations

The gnomAD resource harmonizes genome sequencing data from a variety of large-scale sequencing projects worldwide (Karczewski et al., 2020). The v3.1.2 data set (GRCh38/hg38) spans 76,156 genomes from unrelated individuals of diverse ancestries. In this study, we calculated the allele frequencies of the known and predicted thyroid cancer oncogenic variome belonging to nine human populations worldwide such as African (n = 20,744), Amish (n = 456), Latino (n = 7,647), Ashkenazi Jewish (n = 1,736), East Asian (n = 2,604), European Finnish (n = 5,316), European non-Finnish (n = 34,029), Middle Eastern (n = 158), and South Asian (n = 2,419) (Karczewski et al., 2020; Collins et al., 2020).

### Validation strategies

The first validation strategy involved utilizing the thyroid cancer dependency map from the DepMap project (https://depmap.org/portal/), a collaborative initiative between the Broad Institute and the Wellcome Sanger Institute (Tsherniak et al., 2017). Cancer cells frequently harbor multiple genetic or epigenetic alterations, resulting in specific vulnerabilities that are absent in normal cells. Although the genomic landscape of cancer has been extensively studied, our understanding of the biological impact of these alterations on tumor-specific vulnerabilities remains limited (Baylin and Jones, 2016; Zhang et al., 2024). This knowledge gap impedes the effective application of precision medicine in clinical practice. To address this issue, the primary objective of the DepMap project is to develop a comprehensive preclinical reference map that links tumor features to tumor dependencies, thereby expediting the advancement of precision treatments.

To validate the actionability of prioritized thyroid cancer genes/proteins with oncogenic variants, the DepMap project conducted systematic loss-of-function screens in well-characterized thyroid cancer cell lines, representing the tumor’s heterogeneity. Clustered Regularly Interspaced Short Palindromic Repeats (CRISPR)-based functional genomic screening data from the DepMap Public 24Q4 dataset were employed to identify gene dependencies in thyroid cancer (Tsherniak et al., 2017). This technology systematically knocks out genes across 588 cancer cell lines, including the 11 thyroid cancer cell lines (Cancer Cell Line Encyclopedia Consortium and Genomics of Drug Sensitivity in Cancer Consortium, 2015). The resulting gene dependency scores were processed using the Chronos algorithm, an advanced computational tool that models the effects of CRISPR knockouts while accounting for variables such as cell growth rates and guide RNA efficiency (Dempster et al., 2021). The dependency scores were analyzed to uncover genetic vulnerabilities and identify potential therapeutic targets specific to thyroid cancer. In this scoring system, lower values indicate higher gene essentiality. A score of 0 denotes a non-essential gene, while a score of ≤ −1 corresponds to the median dependency of all common essential genes. This approach provides high-resolution insights into gene dependencies, facilitating the identification of novel targets for precision oncology strategies in thyroid cancer (López-Cortés et al., 2020b; Tsherniak et al., 2017).

The second validation strategy to determine the relevance of the genes/proteins prioritized in our analysis involved evaluating the frequency of genomic alterations in a cohort of thyroid cancer human patients. These findings were then compared with a set of genes and proteins not associated with cancer with an OncoScore <20 (Piazza et al., 2017). To achieve this, we retrieved genomic, transcriptomic, and proteomic alteration data from the PanCancer Atlas, which is part of The Cancer Genome Atlas (TCGA) consortium (Hoadley et al., 2018; Huang et al., 2018; Armendáriz-Castillo et al., 2020). Genomic alterations, including driver mutations, structural variants, copy number variants (CNVs), mRNA upregulation, mRNA downregulation, protein upregulation, and protein downregulation, were analyzed in a cohort of 496 thyroid cancer patients. According to the Genomics Data Commons of the National Cancer Institute (https://portal.gdc.cancer.gov/) and cBioPortal (http://www.cbioportal.org/) (Cerami et al., 2012; Gao et al., 2013), driver mutations were identified through whole-exome sequencing. mRNA upregulation and downregulation were analyzed using RNA sequencing V2 RSEM, where tumor sample expression Z-scores were compared with the distribution of log-transformed mRNA expression in adjacent normal samples (Li and Dewey, 2011). CNV amplifications and deep deletions were identified using GISTIC2.0 (Mermel et al., 2011). Protein upregulation and downregulation were measured using reverse-phase protein arrays, with tumor sample expression Z-scores compared against adjacent normal samples. Finally, a Bonferroni correction (P < 0.001) was applied to perform a multiple comparison test. This test assessed alteration frequencies across three groups: all thyroid cancer driver genes and proteins, the prioritized thyroid cancer driver genes and proteins, and non-cancer driver genes and proteins.

The third validation strategy aimed to assess the ligandability of the thyroid cancer genes/proteins prioritized in our study. This analysis was conducted using canSAR (http://cansar.icr.ac.uk), a comprehensive knowledgebase designed to facilitate drug discovery. canSAR integrates extensive datasets from genomics, proteomics, pharmacology, drugs, and chemicals, alongside structural protein information and protein networks (Mitsopoulos et al., 2021). The resource encompasses the entire human proteome, comprising 20,375 sequences derived from the UniProt Swiss-Prot database (UniProt Consortium, 2019). Additionally, canSAR provides a detailed structure-based ligandability assessment, evaluating over 4.5 million protein cavities (Gingrich et al., 2024).

Protein ligandability is quantified using a chemistry-based scoring system, categorized into four levels: low (0%–24%), indicating the protein is unlikely to be a successful drug target; moderate (25%–49%), suggesting a moderate probability of druggability; high (50%–74%), indicating a good likelihood of druggability; and very high (75%–100%), signifying that the protein is highly druggable and often prioritized for drug development due to its strong potential for successful drug binding. Using this resource, we retrieved both the chemistry-based scores and cancer-specific scores to validate the ligandability of the prioritized thyroid cancer genes and proteins, determining their potential as viable therapeutic targets (Mitsopoulos et al., 2021; Gingrich et al., 2024).

### Therapeutic actionable genomic alterations and *in silico* drug prescription

Another approach in CGI is the *in silico* drug prescription, which involves identifying therapeutic actionable genomic alterations for drug response in tumors and organizing them based on their level of clinical relevance (Muiños et al., 2021). This method uses two resources, the Cancer Biomarker database (Dienstmann et al., 2015) and the Cancer Bioactivities database (Tamborero et al., 2018), to explore the association between the oncogenic variome genomic and drug response. As such, we performed an *in silico* analysis to determine the druggability of thyroid cancer driver proteins carrying known and predicted oncogenic variants. This analysis allowed us to identify the most relevant therapeutic strategies based on precision oncology.

### Drugs involved in clinical trials

The Open Targets Platform (https://www.targetvalidation.org) displays a comprehensive and robust data integration system for access to and visualization of potential therapeutic targets and drugs involved in clinical trials associated with cancer (Carvalho-Silva et al., 2019; Ochoa et al., 2021). Additionally, the Drug Repurposing Hub (https://www.broadinstitute.org/drug-repurposing-hub) is a bioinformatics resource that allowed us to identify the mechanism of action of the US Food and Drug Administration (FDA)-approved drugs (Corsello et al., 2017). Lastly, we created Sankey plots to better understand which drugs are involved in the most advanced phases (III and IV) of thyroid cancer clinical trials.

## Results

### Incidence and mortality of thyroid cancer

**FIGURE 1:**
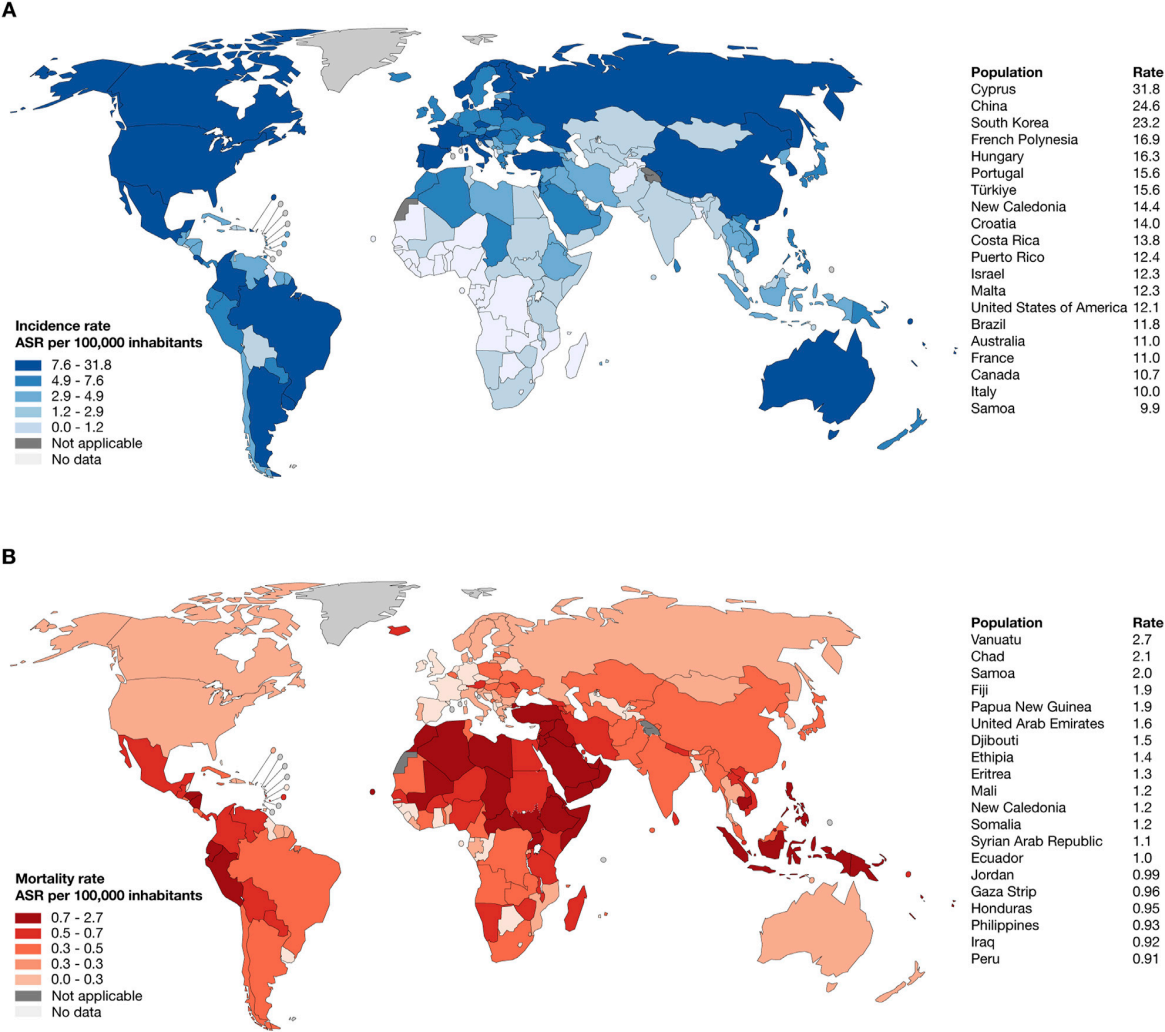
Epidemiology of thyroid cancer. **(A)** Heatmap and ranking of estimated age-standardized incidence rate of thyroid cancer per 100,000 inhabitants worldwide. **(B)** Heatmap and ranking of estimated age-standardized mortality rate of lung cancer per 100,000 inhabitants worldwide.

According to the WHO and GLOBOCAN, the top ten countries worldwide with the highest estimated age-standardized incidence rates of thyroid cancer per 100,000 inhabitants were Cyprus (31,8), China (24,6), South Korea (23,2), French Polynesia (16,9), Hungary (16,3), Portugal (15,6), Türkiye (15,6), New Caledonia (14,4), Croatia (14), and Costa Rica (13,8) (Figure 1A; Supplementary Table S1); meanwhile, the top ten countries worldwide with the highest estimated age-standardized mortality rate were Vanuatu (2,7), Chad (2,1), Samoa (2), Fiji (1,9), Papua New Guinea (1,9), United Arab Emirates (1,6), Djibouti (1,5), Ethiopia (1,4), Eritrea (1,3), and Mali (1,2) (Figure 1B; Supplementary Table S2) (Bray et al., 2024).

### Thyroid cancer driver genes

We have retrieved 40 thyroid cancer driver genes from the intOGen framework (Martínez-Jiménez et al., 2020). Of them, 17 (43%) were metastatic genes (Zheng et al., 2018), 15 (38%) were oncogenes and tumor suppressor genes (Sondka et al., 2018), 10 (25%) were kinase genes (Manning et al., 2002; Eid et al., 2017), 5 (13%) were cancer immunotherapy genes (Patel et al., 2017), 5 (13%) encoded RNA-binding proteins (Hentze et al., 2018), 4 (10%) were DNA-repair genes (Lange et al., 2011; Wood et al., 2001), and 1 (3%) were cell cycle genes (Bar-Joseph et al., 2008) (Supplementary Table S3).

### Identification of the thyroid oncogenic variome and its deleteriousness scores

**FIGURE 2:**
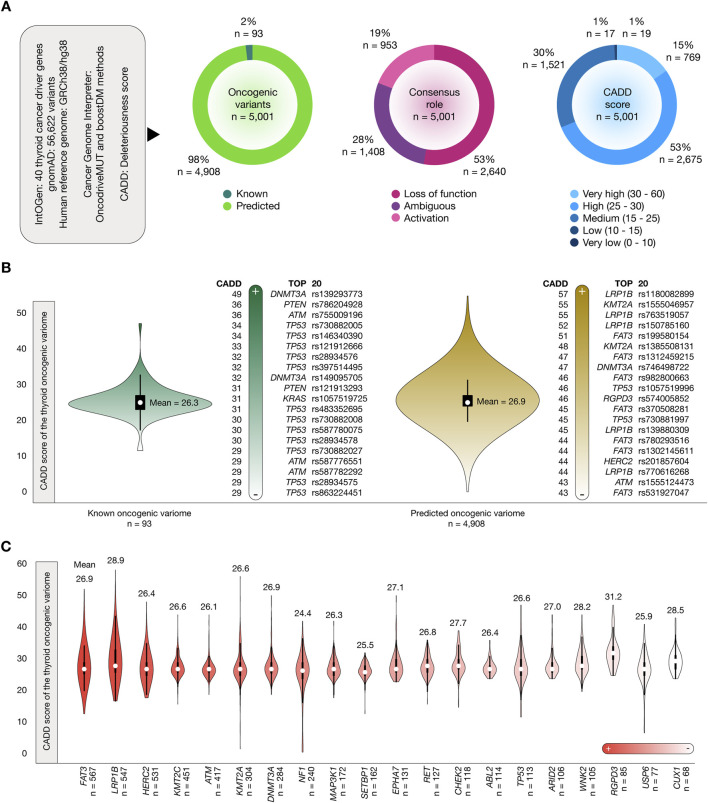
Thyroid cancer driver genes, oncogenic variants, and CADD deleteriousness scores. **(A)** Features of thyroid cancer driver genes, oncogenic variants, consequence type, and CADD deleteriousness scores. **(B)** Bean plots of CADD deleteriousness scores of the thyroid oncogenic variome, and ranking of annotated and predicted oncogenic variants with the highest CADD deleteriousness scores. **(C)** Ranking of the thyroid cancer driver genes with the highest number of oncogenic variants and their mean CADD deleteriousness scores.

Figure 2A shows the results of the OncodriveMUT and boostDM analyzes to identify the oncogenic variome of 40 thyroid cancer driver genes by using the GRCh38/hg38 human reference genome. After the analysis of 56,622 variants, we identified 5,001 oncogenic variants. Of them, 93 (2%) were known and 4,908 (98%) were predicted. The consensus role showed that 2,640 (53%) variants produced a loss of function and 953 (19%) produced protein activation. Regarding the deleteriousness score, 769 (15%) oncogenic variants had very high CADD scores, and 2,675 (53%) oncogenic variants had high CADD scores. Additionally, the consequence type showed that 4,602 (92%) were missense variants, 228 (5%) were stop gained variants, 76 (1.5%) were splice acceptor variants, 61 (1%) were splice donor variants, 33 (0.7%) were splice region variants, and 1 (0.02%) was a start lost variant (Supplementary Table S4). Figure 2B shows violin plots and ranking of CADD scores of the known and predicted oncogenic variome related to thyroid cancer driver genes. The mean CADD score of the known oncogenic variants was 26.3. The known oncogenic variant with the highest CADD score was *DNMT3A* rs139293773 (score = 49). The mean CADD score of the predicted oncogenic variants was 26.9. The predicted oncogenic variant with the highest CADD score was *LRP1B* rs1180082899 (score = 57). The ranking of the 5,001 oncogenic variants is fully detailed in Supplementary Table S5. Finally, Figure 2C details the number of known and predicted oncogenic variants per thyroid cancer driver gene. Genes with the highest number of oncogenic variants were *FAT3* (n = 567), *LRP1B* (n = 547), and *HERC2* (n = 531).

### Protein-protein interactome network

**FIGURE 3:**
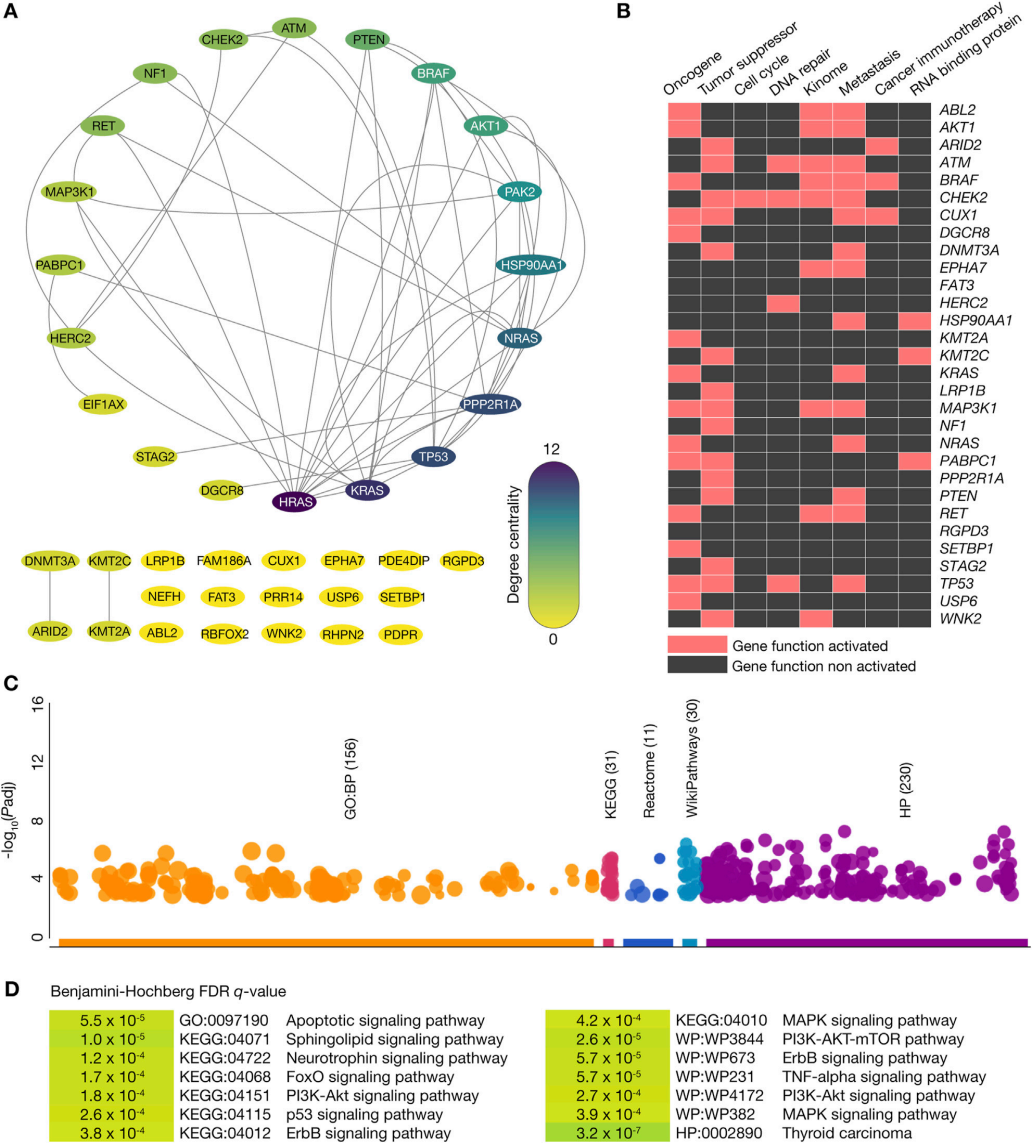
Protein-protein interactome and functional enrichment analysis. **(A)** Protein-protein interactome of the thyroid cancer driver genes where the top ten proteins with the highest degree of centrality were HRAS, KRAS, TP53, PPP2R1A, NRAS, HSP90AA1, PAK2, AKT1, BRAF, and PTEN. **(B)** Heatmap of thyroid cancer driver genes with oncogenic variants being part of oncogenes, tumor suppressor genes, cell cycle genes, DNA repair genes, kinome, metastatic genes, cancer immunotherapy genes, and genes encoding RNA-binding proteins. **(C)** Manhattan plot of the most significant GO biological processes, KEGG signaling pathways, Reactome signaling pathways, WikiPathways, and Human Phenotype Ontology annotations. **(D)** Most significant (Benjamini–Hochberg FDR q-value <0.001) GO biological processes, KEGG signaling pathways, Reactome signaling pathways, WikiPathways, and Human Phenotype Ontology annotations where the thyroid cancer driver genes with oncogenic variants were involved.

The PPi network was generated to better understand the connectivity between thyroid cancer driver proteins with high-confidence interactions (cutoff = 0.9). This network comprised 40 nodes (100%) and 50 high-confidence edges, as shown in Figure 3A. Among the 40 nodes, 30 (75%) represented thyroid cancer driver genes carrying oncogenic variants, while 10 (25%) did not carry oncogenic variants. To further analyze the network’s structure, the degree centrality of the nodes was calculated, identifying the top ten thyroid cancer driver proteins with the highest degree of connectivity: HRAS (12), KRAS (10), TP53 (9), PPP2R1A (9), NRAS (8), HSP90AA1 (7), PAK2 (6), AKT1 (5), BRAF (5), and PTEN (4). These results provide insights into the key proteins and their interactions within the thyroid cancer driver protein network, emphasizing the potential roles of these central nodes in thyroid cancer progression.

### Functional enrichment analysis

Figure 3B displays a heatmap of the 30 thyroid cancer driver genes carrying known and predicted oncogenic variants involved in several tumorigenic processes. We identified 15 tumor suppressor genes, 14 metastatic genes, 12 oncogenes, 8 kinome genes, 4 DNA repair genes, 3 genes encoding RNA binding proteins, 2 cancer immunotherapy genes, and 1 cell cycle gene. Using the g:Profiler bioinformatics tool (Raudvere et al., 2019), we performed functional enrichment analysis on these 29 thyroid cancer driver genes with known and predicted oncogenic variants and identified 156 GO biological processes (The Gene Ontology Consortium, 2021), 31 KEGG signaling pathways (Kanehisa and Goto, 2000), 11 Reactome signaling pathways (Fabregat et al., 2016), 30 Wikipathways (Slenter et al., 2018), and 230 HP ontologies (Köhler et al., 2021), as shown in the Manhattan plot of Figure 3C. Subsequently, we found the most significant (Benjamini–Hochberg, FDR *q* < 0.001) annotations related to thyroid cancer to be apoptotic (GO:0097190), sphingolipid (KEGG:04071), neurotrophin (KEGG:04722), FoxO (KEGG:04068), p53 (KEGG:04115), ErbB (KEGG:04012), MAPK (KEGG:04010), PI3K-AKT-mTOR (WP:WP3844), and TNF-α (WP:WP231) signaling pathways. Finally, we observed that the thyroid carcinoma annotation was significant as a human phenotype ontology (HP:0002890) (Figure 3D; Supplementary Table S6).

### Deleteriousness scores and allele frequencies across human populations

**FIGURE 4:**
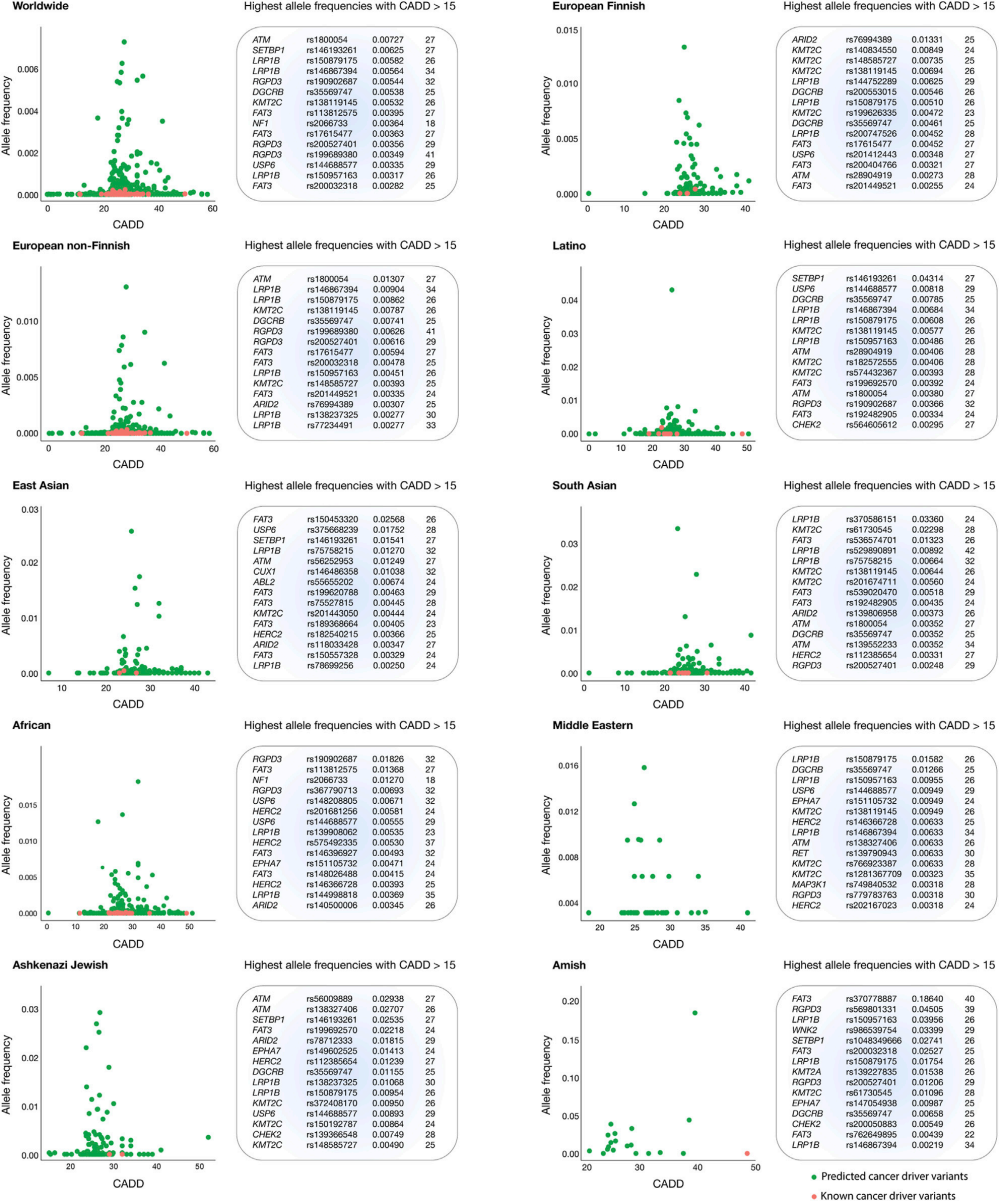
Thyroid cancer oncogenic variants with the highest allele frequencies and CADD deleteriousness scores. Scatter plots and ranking of the annotated and predicted oncogenic variants with the highest allele frequencies and CADD deleteriousness scores from the European Finnish, European non-Finnish, Latino, East Asian, South Asian, African, Middle Eastern, Ashkenazi Jewish, and Amish populations.

Figure 4 presents scatter plots that highlight oncogenic variants with the highest allele frequencies and the most deleterious CADD scores per human population. The Amish population had the highest mean CADD score (29.9), followed by Middle Eastern (27.3), Latino (27.0), European Finnish (27.0), European non-Finnish (26.9), East Asian (26.9), African (26.9), Ashkenazi Jewish (26.9), and South Asian (26.7) populations.

Globally, the top five oncogenic variants with the highest allele frequencies were *ATM* rs1800054 (0.00727), *SETBP1* rs146193261 (0.00625), *LRP1B* rs150879175 (0.00582), *LRP1B* rs146867394 (0.00564), and *RGPD3* rs190902687 (0.00544. The *ARID2* rs76994389 oncogenic variant displayed the highest allele frequency in the European Finnish population (0.01331); *ATM* rs1800054 in the European non-Finnish population (0.01307); *SETBP1* rs146193261 in the Latino population (0.04314); *FAT3* rs150453320 in the East Asian population (0.02568); *LRP1B* rs370586151 in the South Asian population (0.03360); *RGPD3* rs190902687 in the African population (0.01826); *LRP1B* rs150879175 in the Middle Eastern population (0.01582); *ATM* rs56009889 in the Ashkenazi Jewish population (0.02938); and *FAT3* rs370778887 in the Amish population (0.18640). Finally, a complete ranking of oncogenic variants with the highest allele frequencies and CADD scores per human population is provided in Figure 4 and Supplementary Table S5.

### Validation strategies

**FIGURE 5:**
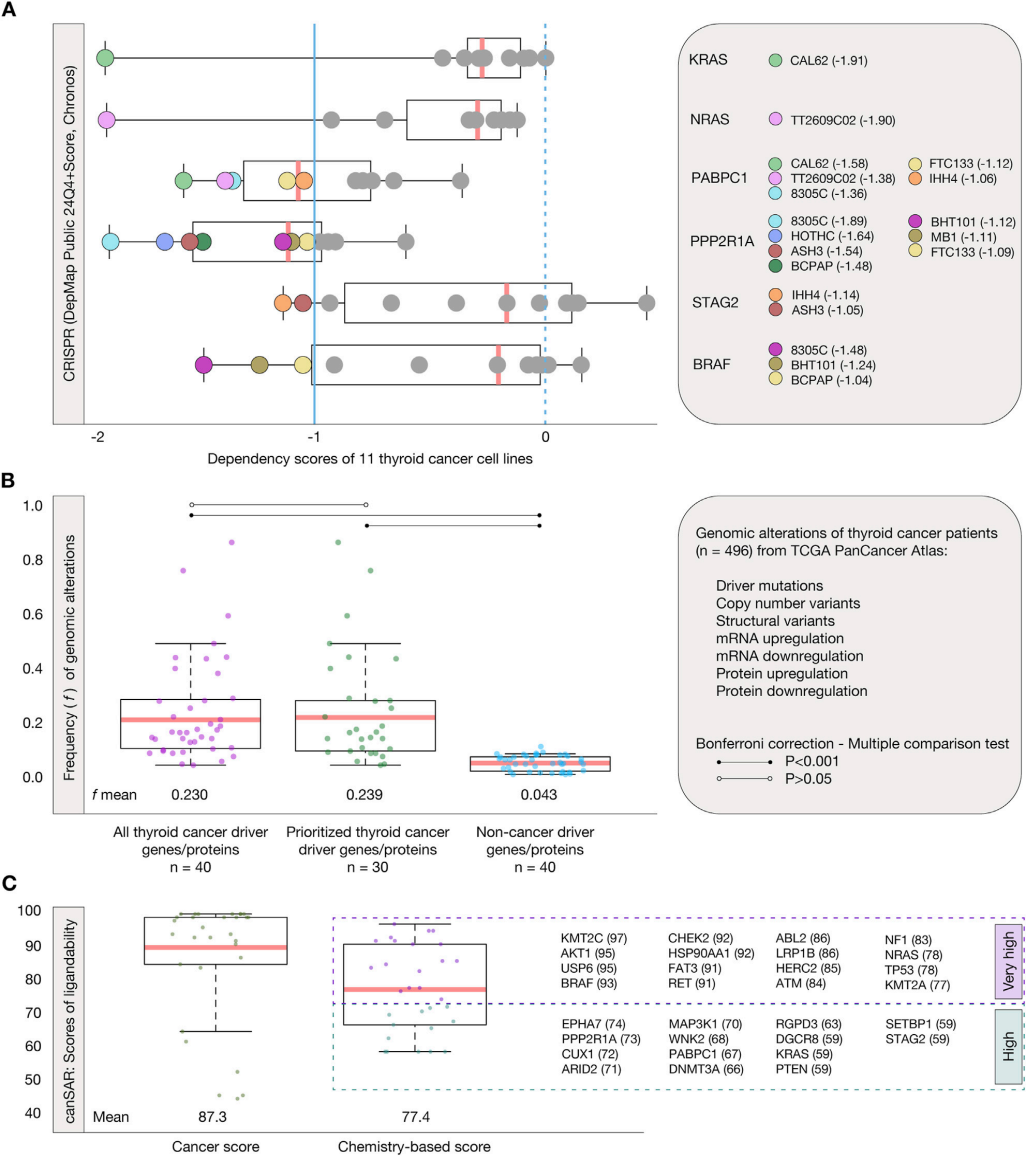
Validation strategies. **(A)** Thyroid cancer dependency map analyzed through CRISPR and the Chronos algorithm. This analysis identifies 19 dependencies of the KRAS, NRAS, PABPC1, PPP2R1A, STAG2, and BRAF into 11 cell lines. The essential proteins have a dependency score < −1. **(B)** Genomic, transcriptomic, and proteomic alterations of 496 thyroid cancer patients from the TCGA PanCancer Atlas. Boxplots are shown to demonstrate the significant statistical difference of alterations between the 30 prioritized thyroid cancer driver genes/proteins and non-cancer driver genes (Bonferroni correction, P < 0.001). **(C)** Ligandability analysis through canSAR. Boxplots are shown to demonstrate the cancer score and chemistry-based score of the 30 prioritized thyroid cancer driver genes/proteins. The analysis reveals that 16 (%) of these proteins have very high ligandability scores (75–100), while 14 have high ligandability scores (50–75).

The first validation strategy consisted in identifying genes/proteins essential for thyroid cancer cell proliferation and survival by performing systematic loss-of-function screens in 11 well-annotated cell lines, as outlined in the thyroid cancer dependency map from the DepMap project (Tsherniak et al., 2017). Figure 5A presents boxplots showing the distribution of dependency scores for 6 thyroid cancer driver genes/proteins, calculated using the Chronos algorithm (DepMap Public 24Q4+Score, Chronos). Our analysis identified 19 gene/protein dependencies with scores ≤ −1 across the 6 driver genes in thyroid cancer cell lines. Specifically, KRAS showed a dependency score of −1.91 in the CAL62 cell line, while NRAS exhibited a dependency score of −1.90 in the TT2609C02 cell line. PABPC1 demonstrated five dependencies, with scores of −1.58 (CAL62), −1.38 (TT2609C02), −1.36 (8305C), −1.12 (FTC133), and −1.06 (IHH4). PPP2R1A exhibited seven dependencies, with scores of −1.89 (8305C), −1.64 (HOTHC), −1.54 (ASH3), −1.48 (BCPAP), −1.12 (BHT101), −1.11 (MB1), and −1.09 (FTC133). STAG2 showed two dependencies with scores of −1.14 (IHH4) and −1.05 (ASH3), while BRAF displayed three dependencies with scores of −1.48 (8305C), −1.24 (BHT101), and −1.04 (BCPAP) (Supplementary Table S7).

The second validation strategy involved calculating the frequency of alterations, including driver mutations, structural variants, CNVs, mRNA upregulation, mRNA downregulation, protein upregulation, and protein downregulation, in a cohort of 496 thyroid cancer patients from the TCGA PanCancer Atlas (Figure 5B; Supplementary Table S8). The set of 40 thyroid cancer driver genes/proteins had a mean alteration frequency of 0.230, while our 30 prioritized thyroid cancer driver genes/proteins exhibited a mean frequency of 0.239. In comparison, the set of 40 non-cancer driver genes/proteins (OncoScore <20) showed a significantly lower mean frequency of 0.043. A Bonferroni correction (P < 0.001) was applied to perform a multiple comparison test among these three groups. Our prioritized genes/proteins did not show a significant difference compared to the entire set of thyroid cancer driver genes/proteins. However, they exhibited a statistically significant difference when compared to the non-cancer driver genes (P < 0.001). These results demonstrate that the prioritized genes/proteins, which are altered in healthy individuals worldwide, also exhibit high frequencies of alterations in a cohort of thyroid cancer patients.

The third validation strategy focused on evaluating the ligandability of the thyroid cancer genes/proteins prioritized in our study. Ligandability refers to a protein’s ability to efficiently bind to a drug, a key factor in identifying and prioritizing effective targets for drug development. Proteins with high ligandability are more likely to enable the development of highly specific drugs, reducing time and cost in pharmaceutical development (Wang et al., 2019; López-Cortés et al., 2024). This analysis was performed using canSAR, a comprehensive knowledgebase dedicated to drug discovery that provides an extensive structure-based ligandability assessment (Mitsopoulos et al., 2021; Gingrich et al., 2024). From canSAR, we retrieved both the chemistry-based scores and the cancer-specific scores for the 30 previously prioritized thyroid cancer driver proteins. The mean chemistry-based score of these proteins was 77.4, while the mean cancer-specific score was 87.3. Our results revealed that 16 (53.3%) exhibited very high ligandability (scores ranging from 75 to 100), and 14 (47.7%) showed high ligandability (scores ranging from 50 to 74) (Figure 5C; Supplementary Table S9). The identification of proteins with high and very high ligandability underscores their potential as effective therapeutic targets for thyroid cancer.

### *In silico* drug prescription targeting therapeutic actionable genomic alterations

**FIGURE 6:**
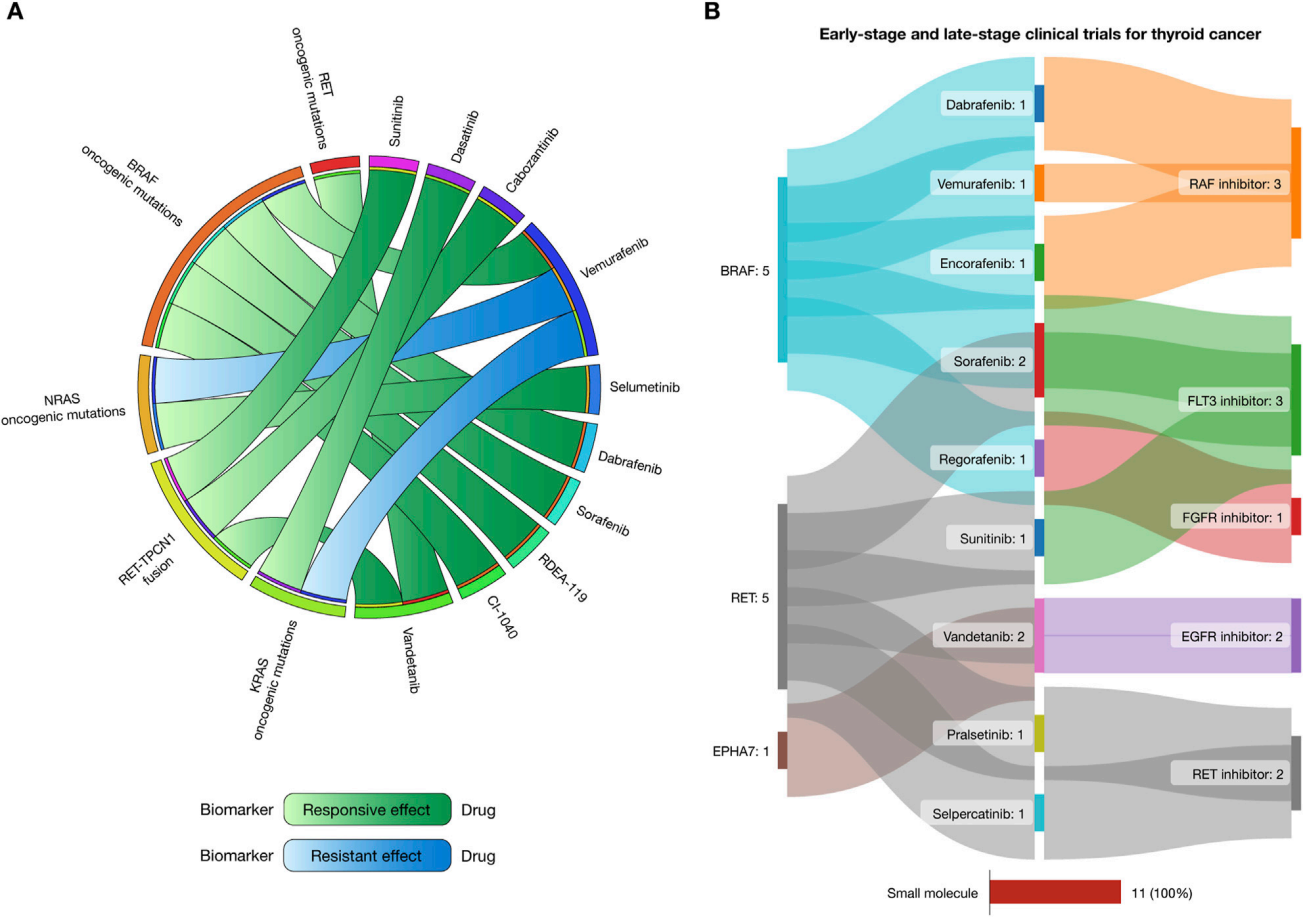
Landscape of therapeutic strategies based on precision oncology. **(A)** Circos plot showing *in silico* drug prescriptions of responsive and resistant effects targeting thyroid cancer actionable genomic alterations. **(B)** Sankey plot of early-stage and late-stage clinical trials for gastric cancer connecting therapeutic targets, drugs, and mechanisms of action.

Putative biomarkers for drug response and resistance in thyroid cancer treatments, as identified from the Cancer Biomarker Database, are presented as a circos plot in Figure 6A (Dienstmann et al., 2015). Patients with RET oncogenic mutations respond well to vandetanib. Those with BRAF mutations show responsiveness to dabrafenib, sorafenib, RDEA-119, and CI-1040. NRAS mutations respond well to selumetinib but exhibit resistance to vemurafenib. Similarly, KRAS mutations respond to dasatinib but are resistant to vemurafenib. Lastly, individuals with RET-TPCN1 fusions respond to sunitinib, cabozantinib, and vandetanib (Supplementary Table S10).

### Drugs involved in clinical trials

The Open Targets Platform provides updates on the progression of clinical trials targeting proteins implicated in cancer (Echeverría-Garcés et al., 2024a; 2024b; Ochoa et al., 2023), while the Drug Repurposing Hub details the mechanism of action of the FDA-approved drugs (Corsello et al., 2017). Figure 6B illustrates a Sankey plot representing 11 clinical trial events where small molecules were involved in early-stage phases (I and II) and late-stage phases (III and IV) for thyroid cancer. These trials involve three targetable proteins (including BRAF, RET, and EPHA7), and 9 small molecule drugs with 5 mechanisms of action. Dabrafenib, vemurafenib, and encorafenib were RAF inhibitors, sorafenib and sunitinib were FLT3 inhibitors, regorafenib was an FGFR inhibitor, vandetanib was an EGFR inhibitor, and pralsetinib and selpercatinib were RET inhibitors (Supplementary Table S11).

## Discussion

Thyroid cancer, one of the most prevalent endocrine malignancies, exhibits substantial heterogeneity in its incidence, progression, and response to treatment. This variability arises a complex interplay of genetic, epigenetic, and environmental factors (Hanahan, 2022; Hanahan and Weinberg, 2011; Ocaña-Paredes et al., 2024; Singh et al., 2021). Such diversity underscores the urgent need for comprehensive strategies to identify actionable genomic alterations, validate therapeutic targets, and advance precision oncology approaches tailored to individual and population-specific needs (Echeverría-Garcés et al., 2024a; Echeverría-Garcés et al., 2024b; Tan et al., 2021). This study addresses these challenges through an integrated, multi-step analysis, leveraging advanced computational tools and diverse datasets to enhance our understanding of thyroid cancer biology and therapeutic opportunities.

Our analysis of GLOBOCAN data revealed significant disparities in thyroid cancer incidence and mortality rates worldwide. High incidence rates were observed in countries such as Cyprus, China, and South Korea, while mortality rates were disproportionately higher in nations like Vanuatu and Chad (Bray et al., 2024). These variantions reflect the interplay of genetic predisposition, healthcare access, environmental exposures, and diagnostic practices. Addressing these disparities is critical for guiding resource allocation and implementing targeted public health interventions, particularly in high-mortality regions with limited healthcare infrastructure (Lyu et al., 2024).

Identifying driver genes is fundamental to understanding tumorigenesis and developing therapeutic targets. Using the intOGen pipeline, 40 thyroid cancer driver genes were identified and categorized by function, including metastatic genes, oncogenes, tumor suppressor genes, and DNA repair genes (Martínez-Jiménez et al., 2020). Analysis of 56,622 single nucleotide and insertion/deletion variants across these genes uncovered 5,001 known and predicted oncogenic variants, with *FAT3*, *LRP1B*, *HERC2*, *KMT2C*, *ATM*, *KMT2A*, *DNMT3A*, *NF1*, *MAP3K1*, and *SETBP1* among the most recurrently altered. Deleteriousness scores calculated using CADD revealed several variants with very high scores, including those in *RGPD3*, *LRP1B*, and *RET*, emphasizing their critical roles in thyroid cancer progression and therapeutic potential (Rentzsch et al., 2019; Schubach et al., 2024).

The PPi network highlighted the centrality and connectivity of critical thyroid cancer driver proteins, including HRAS, KRAS, and TP53, which act as hubs in tumorigenic signaling pathways (Doncheva et al., 2019). This network analysis underscores the importance of these proteins as potential therapeutic targets and their broader roles in influencing tumor progression through interactions with other proteins (Porta-Pardo et al., 2015). On the other hand, functional enrichment analysis of the 30 prioritized driver genes/proteins identified key biological pathways implicated in thyroid cancer (Reimand et al., 2019). Pathways such as FoxO, p53, ErbB, MAPK, PI3K-AKT-mTOR, and TNF-α signaling were significantly enriched, highlighting their roles in tumorigenesis and as therapeutic targets. These findings provide a foundation for exploring pathway-specific inhibitors and combination therapies to address pathway redundancies and resistance mechanisms (Tomuleasa et al., 2024).

Three validation strategies confirmed the therapeutic relevance of the 30 prioritized thyroid cancer driver genes/proteins. The first utilized CRISPR-based functional genomics through loss-of-function screens from the DepMap project (Tsherniak et al., 2017). This approach identified significant dependencies on key genes/proteins such as KRAS, NRAS, PABPC1, PPP2R1A, STAG2, and BRAF in thyroid cancer cell lines, underscoring their potential as critical therapeutic targets. The second strategy analyzed genomic alteration frequencies in a cohort of 496 thyroid cancer patients from the TCGA PanCancer Atlas, demonstrating significantly higher alteration frequencies in prioritized genes/proteins compared to non-cancer-associated genes/proteins, further validating their clinical utility (Cerami et al., 2012; Gao et al., 2013). The third strategy assessed ligandability using canSAR, identifying 16 proteins with very high ligandability scores and 14 with high scores. These findings reinforce the suitability of these proteins as druggable targets, enhancing the specificity and efficacy of therapies while reducing drug development costs (Gingrich et al., 2024).

A significant challenge in advancing thyroid cancer treatment is the limited diversity in genomic research. Cancer genomic studies have predominantly focused on Caucasian populations, which restricts the generalizability of pharmacogenomic findings to other ethnic groups (Guerrero et al., 2018; García-Cárdenas et al., 2024). This lack of diversity can lead to disparities in treatment efficacy, as certain genetic variants may be more prevalent or behave differently in various populations. Expanding research to encompass a wider demographic could allow for the identification of population-specific variants and enable the refinement of treatment protocols to address genetic variability (Zavala et al., 2021; Spratt et al., 2016). In this context, our study identified 219 pathogenic alterations in the European Finnish population, 1,688 in European non-Finnish, 738 in Latino, 447 in East Asian, 447 in South Asian, 1,817 in African, 51 in Middle Eastern, 157 in Ashkenazi Jewish, and 20 in the Amish population. Notably, the most frequent pathogenic variant in the European Finnish population was *ARID2* rs76994389 (0.01331), in European non-Finnish was *ATM* rs1800054 (0.01307), in Latino was *SETBP1* rs146193261 (0.04314), in East Asian was *FAT3* rs150453320 (0.02568), in South Asian was *LRP1B* rs370586151 (0.03360), in African was *RGPD3* rs190902687 (0.01826), in Middle Eastern was *LRP1B* rs370586151 (0.01582), in Ashkenazi Jewish was *ATM* rs56009889 (0.02938), and in Amish was *FAT3* rs370778887 (0.18640). A deep understanding of these variants is critical for devising preventive strategies and tailoring effective treatment options for lung cancer patients in these populations.

Emerging technologies such as artificial intelligence and *in silico* modeling are becoming invaluable in precision oncology (Yumiceba et al., 2020; López-Cortés et al., 2021a; 2022b; Pérez-Villa et al., 2023). These tools facilitate high-throughput screening of potential therapeutic compounds, analysis of drug response, and prediction of resistance mechanisms, all of which are essential for refining treatment protocols (López-Cortés et al., 2020b; López-Cortés et al., 2020a; López-Cortés et al., 2018; López-Cortés et al., 2024). By identifying common alterations across different populations, our research has integrated these findings with *in silico* drug recommendations (Tamborero et al., 2018) and data from early- and late-stage clinical trials (Ochoa et al., 2021), thus enhancing the ability to detect significant oncogenic alterations in thyroid cancer patients. This approach supports the development of more tailored and effective treatment plans for thyroid cancer patients across diverse ethnic backgrounds. The use of drug inhibitors (sunitinib, dasatinib, cabozantinib, vemurafenib, selumetinib, dabrafenib, sorafenib, RDEA-119, CI-1040, vandetanib, encorafenib, regorafenib, pralsetinib, and selpercatinib) targeting *RET*, *BRAF*, *NRAS*, *KRAS*, and *EPHA7* mutations has shown efficacy in reducing tumor progression in thyroid cancer patients. However, as with other targeted therapies, resistance remains a considerable obstacle. Exploring combination therapies that target multiple pathways simultaneously may mitigate resistance and improve patient outcomes (Cabanillas et al., 2019; Zhong et al., 2021).

Pharmacogenomic research in thyroid cancer focuses on personalizing treatment by evaluating how genetic variations affect drug response and toxicity (Quinones et al., 2014; Ocaña-Paredes et al., 2024; Salas-Hernández et al., 2023). While pharmacogenomic guidelines are emerging, standardized recommendations remain underdeveloped. Establishing comprehensive guidelines is critical for optimizing drug dosing, reducing adverse effects, and improving patient adherence to treatments (López-Cortés et al., 2020c).

Advancing precision oncology in thyroid cancer requires expanding research to include diverse populations, integrating bioinformatics tools, and developing comprehensive pharmacogenomic guidelines. Building on the success of targeted therapies in other cancers, ongoing clinical trials should continue exploring novel therapeutic targets and drug combinations. Strengthening international collaboration could expedite the creation of multi-ethnic genomic databases, fostering more inclusive and representative research. Precision oncology holds significant promise for transforming thyroid cancer treatment. Addressing gaps in genomic diversity, overcoming therapeutic resistance, and leveraging advanced technologies will enable the development of tailored therapies, improving outcomes for patients across diverse populations. This approach aligns with the principles of personalized medicine and emphasizes equity in cancer treatment globally.

## Data Availability

The original contributions presented in the study are included in the article/Supplementary Material, further inquiries can be directed to the corresponding author.
